# Genetic Risk Factors in Idiopathic and Non-Idiopathic Interstitial Lung Disease: Similarities and Differences

**DOI:** 10.3390/medicina60121967

**Published:** 2024-11-29

**Authors:** Stefania Cerri, Elisa Manzini, Ottavia Nori, Lucia Pacchetti, Laura Rossi, Maria Giulia Turchiano, Anna Valeria Samarelli, Giulia Raineri, Dario Andrisani, Filippo Gozzi, Bianca Beghè, Enrico Clini, Roberto Tonelli

**Affiliations:** 1 Respiratory Disease Unit, University Hospital of Modena, 41124 Modena, Italy; stefania.cerri@unimore.it (S.C.); maria.turchiano@outlook.it (M.G.T.); andrisani.dario@aou.mo.it (D.A.); fillo.gzz@gmail.com (F.G.); bianca.beghe@unimore.it (B.B.); enrico.clini@unimore.it (E.C.); 2 Laboratory of Experimental Pneumology, Department of Surgical and Medical Science, University of Modena and Reggio Emilia, 41124 Modena, Italy; giuliaraineri@unimore.it; 3 Center for Rare Lung Diseases, University Hospital of Modena, 41124 Modena, Italy; 4 Post Doctoral School in Respiratory Medicine, University of Modena and Reggio Emilia, 41124 Modena, Italy; elisa.manzini92@gmail.com (E.M.); ottavia.nori.on@gmail.com (O.N.); lucia.pacchetti@gmail.com (L.P.); lau.mrossi@gmail.com (L.R.); 5 Respiratory Disease Unit, Hospital of Sassuolo, 41049 Sassuolo, Italy; 6 U.O. Pneumologia, Presidio Ospedaliero di Arco, APSS Provincia Autonoma di Trento, 38062 Trento, Italy; 7 Division of Pneumology, MultiMedica IRCCS, 20099 Milan, Italy; 8 Respiratory Disease Unit, Arcispedale Santa Maria Nuova, 42123 Reggio Emilia, Italy

**Keywords:** idiopathic pulmonary fibrosis, interstitial lung disease, familial pulmonary fibrosis, epigenetics, telomere-related genes, surfactant proteins, MUC5B variants, TOLLIP, non-coding RNAs, DNA methylation, fibrosis pathways, personalized medicine

## Abstract

Recent advances in genetics and epigenetics have provided critical insights into the pathogenesis of both idiopathic and non-idiopathic interstitial lung diseases (ILDs). Mutations in telomere-related genes and surfactant proteins have been linked to familial pulmonary fibrosis, while variants in MUC5B and TOLLIP increase the risk of ILD, including idiopathic pulmonary fibrosis and rheumatoid arthritis-associated ILD. Epigenetic mechanisms, such as DNA methylation, histone modifications, and non-coding RNAs such as miR-21 and miR-29, regulate fibrotic pathways, influencing disease onset and progression. Although no standardized genetic panel for ILD exists, understanding the interplay of genetic mutations and epigenetic alterations could aid in the development of personalized therapeutic approaches. This review highlights the genetic and epigenetic factors driving ILD, emphasizing their potential for refining diagnosis and treatment.

## 1. Introduction

**Figure 1:**
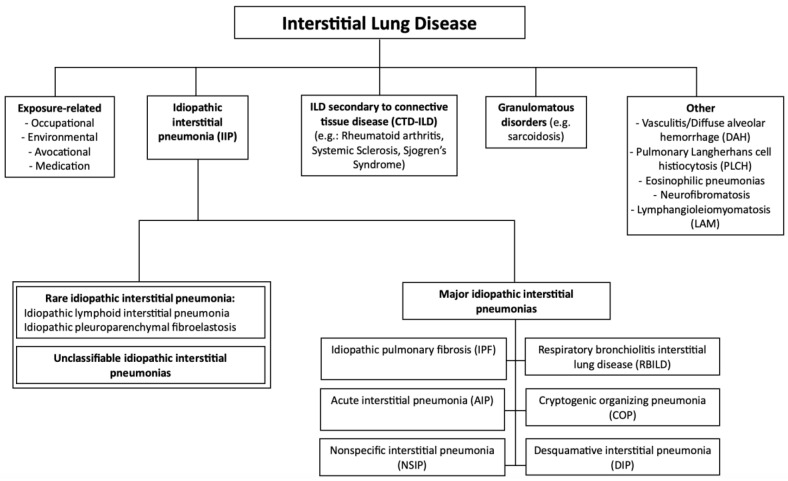
Classification of interstitial lung diseases.

Interstitial lung disease (ILD) encompasses a broad spectrum of pulmonary disorders characterized by inflammation and/or fibrosis within the alveolar interstitium of the lungs [1]. These diseases are rare, with a population prevalence of less than 0.05 percent, but they share common clinical, radiological, and physiological features [2]. The development of pulmonary fibrosis involves multiple risk factors, which can be categorized as environmental and genetic. Environmental factors include advanced age, smoking, respiratory infections, gastroesophageal reflux, and occupational or environmental exposures [3,4]. On the genetic side, specific genetic variants and epigenetic modifications have been implicated in the disease’s pathogenesis [3,5]. The strongest evidence for a genetic predisposition to ILD comes from its clustering in families, particularly in homozygous twins raised apart [5], multigenerational families [6,7], and genetically related relatives [8]. Based on etiology, ILDs can be divided into idiopathic forms (idiopathic interstitial pneumonia, IIP) and those with known causes, such as connective tissue diseases (CTD-ILDs) or secondary to environmental exposures, radiation, or drug use. Among the idiopathic forms, idiopathic pulmonary fibrosis (IPF) is the most prevalent, accounting for 20–50% of all diffuse infiltrative lung diseases. IPF is a progressive fibrosing condition that, without antifibrotic therapy, leads to respiratory failure and death within 3–5 years of diagnosis. According to guidelines, IPF diagnosis requires excluding other known causes of ILD and identifying the high-resolution computed tomography (HRCT) pattern of usual interstitial pneumonia (UIP) or specific combinations of HRCT and histopathology findings from lung tissue samples. Figure 1 illustrates the classification and subdivision of ILD types and subtypes.

Within idiopathic forms of ILD, IPF can be further classified into sporadic, familial, and syndromic forms. Familial IPF (f-IPF), which accounts for 20% of IPF cases, is diagnosed when at least two biological family members exhibit the clinical and histological features of IPF. Familial pulmonary fibrosis (FPF) refers to the presence of any fibrosing ILD in two or more first- or second-degree relatives [8]. Familial forms tend to have an earlier onset and faster progression toward fibrosing ILD compared with sporadic cases. Among relatives with FPF, UIP is the most frequent phenotype; however, more than 50% of affected families exhibit multiple ILD phenotypes, including nonspecific interstitial pneumonia (NSIP), hypersensitivity pneumonitis (HP), and rheumatoid arthritis-associated ILD (RA-ILD) [6]. In contrast, sporadic IPF (s-IPF) has a multifactorial etiology, with environmental factors sometimes interacting with genetic variants. 

Both idiopathic and non-idiopathic forms of ILD share a common genetic background [7,9,10,11]. Genetic mutations, occurring at frequencies below 1% in the general population, differ from common variants, which have frequencies greater than 1%. Mutations in telomere maintenance genes (telomerase reverse transcriptase [TERT], telomerase RNA component [TERC], Poly (A)-specific ribonuclease [PARN], regulator of telomere length 1 [RTEL1]) lead to telomere shortening, accelerated cellular aging, and abnormal epithelial repair [12], while mutations in surfactant protein (SP)-encoding genes (SFTPA1, SFTPA2, SFTPC, ABCA3) are also implicated. On the other hand, variants in the Mucin 5 b (MUC5B) gene, which regulates mucosal defense, and the Toll-interacting protein (TOLLIP) gene also contribute to disease susceptibility. This review highlights the key genetic risk factors identified in ILDs, emphasizing the similarities and differences between idiopathic and non-idiopathic forms. Gene mutations and variants associated with ILDs are summarized in Table 1. Furthermore, epigenetic alterations, including histone modifications, DNA methylation, and non-coding RNA-mediated gene silencing, which play a significant role in ILD pathogenesis, are thoroughly examined.

## 2. Telomere Dysfunction

Telomeres, the terminal regions of eukaryotic chromosomes, consist of tandem DNA repeats and play a vital role in the cell division cycle. During DNA replication, the inability to fully replicate the ends of chromosomes results in the progressive shortening of telomeres over time [3]. To mitigate this, the enzyme telomerase extends telomeres by adding repetitive DNA sequences to the chromosomal ends, thereby preserving their length [13]. Telomerase comprises two main subunits: telomerase reverse transcriptase (TERT), which serves as the catalytic component responsible for telomere elongation, and telomerase RNA component (TERC), a non-coding RNA that provides the structural framework for the telomerase complex. Additionally, RTEL1, a DNA helicase, contributes to maintaining telomere integrity, while PARN, an enzyme involved in mRNA regulation during development, plays a critical role in sustaining telomere length in fibroblasts and facilitating the induction of pluripotent stem cells [14,15]; however, telomerase activity is not universally expressed. It is active in proliferating cells, such as stem cells, progenitors, and precursors, but absent in most terminally differentiated or quiescent cells, such as somatic cells [16].

Mutations in telomere-related genes (TRG) can disrupt the maintenance of telomere length, resulting in progressive shortening. Once this shortening reaches a critical threshold, DNA damage occurs, leading to cellular apoptosis [17]. Telomere length is considered short when it falls below the 10th percentile for a given age group [18]. The measurement of telomere length in peripheral blood cells is often used as a proxy for detecting telomere mutations [19]. Notably, telomere shortening is closely linked to aging and age-related diseases, as it tends to decrease with age [20,21]. Mutations in TRG were initially identified in patients with dyskeratosis congenita (DKC) and later in those suffering from bone marrow failure syndromes, pulmonary fibrosis, and liver disease [13]. Although the exact mechanisms driving ILD pathogenesis remain unclear, there is growing evidence that telomere shortening and genetic instability—hallmarks of early cellular aging—play a key role in the fibrotic process [22,23]. Telomere dysfunction is thought to induce senescence and apoptosis, particularly in alveolar stem cells (alveolar epithelial type 2 cells, or AEC type 2) [24]. This, in turn, contributes to pulmonary fibrosis through two main pathways: first, it impairs the regeneration of functional alveolar tissue [25], and second, it triggers a cytokine cascade that recruits inflammatory cells, including fibroblasts and myofibroblasts [26,27,28].

### 2.1. Telomere Dysfunction in IPF and FPF

IPF accounts for more than 70% of all cases of IIPs [29]. IPF manifests in both sporadic and familial forms, with mutations in telomere-related genes (TRG) identified in both TRG mutations have been found in approximately 25% of f-IPF cases and 1–3% of sporadic cases [30]. Among familial IPF patients with telomere mutations, half have mutations in the TERT or TERC genes [31], while the other half may carry mutations in other telomere-related genes such as RTEL1 or PARN. Mutations in TERT, TERC, PARN, and RTEL1 impair telomerase activity, accelerating telomere shortening (Table 1) [3]. Sporadic IPF is multifactorial, involving environmental risk factors that sometimes interact with variant in numerous genes. These patients show shorter blood leukocyte telomeres—ranging from 5.71% to 68.2% shorter than controls—without the presence of a clear TRG mutation. In sporadic IPF, short telomeres are associated with worse survival outcomes [32], suggesting that the pathways involved in familial disease related to telomere dysfunction (even in the absence of genetic mutations) may also contribute to sporadic forms of the disease [33,34]. Silencing TERT expression results in telomerase inactivation [35]. As a result, mutations in TERT are linked to short telomere syndromes and can present a broad spectrum of clinical phenotypes [36]. Families with TERT mutations tend to have an earlier onset of pulmonary fibrosis, typically occurring between the ages of 60 and 70 [37]. In a study by Alder et al., 45 individuals from 10 families with known TRG mutations were screened. Comparisons between mutation carriers and their non-carrier first-degree relatives revealed that individuals with TRG mutations had significantly shorter telomeres (*p* < 0.0001) [19]. Despite this, identifying TRG mutations in patients with sporadic forms of idiopathic interstitial pneumonia (IIP) remains rare [19]. For example, Tsakari et al. identified only one hTERT mutation among 44 sporadic IPF cases [34]. Armanios et al. evaluated 73 families with familial pulmonary fibrosis (FPF) and found that six of them had shorter telomeres in peripheral blood leukocytes than their peers. These families also carried TERT/TERC heterozygous mutations that code for RNA ligands of the telomerase complex, expressed in an autosomal dominant pattern [31,38]. Mutations in RTEL1 lead to the loss of telomeric repeats, which results in telomere shortening [39]. Telomere lengths in RTEL1 mutation carriers are similar to those in patients with TERT or TERC mutations, particularly in peripheral blood mononuclear cells (PBMCs) [40]. Rare RTEL1 mutations have been associated with familial ILD, especially in patients with short telomeres in PBMCs [40]. The exact mechanism linking PARN mutations to telomere shortening remains unclear [41,42]. In a study by Stuart et al., leukocyte telomere lengths in participants with PARN and RTEL1 mutations were significantly shorter compared with those in unrelated kindreds (*p* < 0.001 for both genes) [43].

### 2.2. Telomere Dysfunction in RA-ILD

RA is a systemic inflammatory and autoimmune disorder, with extra-articular involvement occurring in nearly 50% of patients. The lungs are frequently affected, often presenting as ILD, with the UIP pattern being the most common [44,45]. Despite its prevalence and significant prognostic impact, much remains unknown about the pathogenesis of RA-ILD [46]. ILD is one of the leading causes of mortality in RA, accounting for 10% to 20% of deaths [47]. In fact, mortality rates are notably higher in patients with RA-ILD compared with those without ILD [47]. IPF, FPF, and RA-ILD share common risk factors, including cigarette smoking and male sex [48,49]. Due to these shared factors, it has been hypothesized that these disorders may also share genetic risk factors, such as telomere mutations. In a groundbreaking study, Juge et al. [9] conducted whole exome sequencing (WES) on patients with RA-ILD to investigate the potential involvement of FPF-linked genes. The study identified heterozygous mutations in telomere-maintenance genes in 12 out of 101 RA-ILD patients, specifically in TERT, RTEL1, and PARN. Notably, no mutations were detected in TERC [9]. Importantly, no other clinical manifestations related to telomere syndromes were observed in mutation carriers. Additionally, pulmonary function tests and HRCT chest scans did not show significant differences between carriers and non-carriers; however, the mean age of ILD onset was significantly earlier in patients with TRG mutations compared with those without [9]. These findings confirm that RA-ILD and FPF share genetic risk factors, suggesting common pathogenic pathways. In terms of telomere lengths, chromosomes isolated from circulating leukocytes in telomere-mutation carriers were significantly shorter than those in controls, highlighting the detrimental effects of these mutations on telomere maintenance. Notably, three of the twelve mutation carriers had a family history of ILD, with PARN mutation carriers exhibiting the shortest telomeres, underscoring the crucial role of PARN in telomere maintenance. Furthermore, ILD onset occurred earlier in patients with RTEL1 and TERT mutations compared with controls. These results are consistent with previous studies and support the concept of genetic anticipation, which is commonly observed in telomere-mediated disorders.

### 2.3. Telomere Dysfunction in Chronic HP

HP is a form of ILD that develops following repeated exposure to environmental inhalant antigens. The condition is characterized by alveolitis and granuloma formation and, in some cases, may progress to chronic fibrotic disease [50]. Emerging evidence suggests that telomere dysfunction plays a role in the pathogenesis and prognosis of patients with chronic HP (CHP) [51]; however, the mechanisms driving fibrosis in HP remain poorly understood. In a subset of CHP patients, shorter telomere lengths in peripheral blood leukocytes (PBLs) have been observed, along with the identification of rare variants in telomere-related genes (TRG) [31,37]. CHP patients carrying telomere mutations tend to have more severe lung fibrosis, often presenting with the UIP pattern, and experience worse survival outcomes [10]. Ley and colleagues investigated the prevalence of TRG mutations in patients with CHP. They analyzed two CHP cohorts to identify variants in genes such as TERT, TERC, RTEL1, and PARN. Their findings revealed that a significant number of patients were carriers of TRG variants, which was associated with shorter telomere lengths in PBLs and a markedly reduced transplant-free survival rate [51].

### 2.4. Telomere Dysfunction in Other ILDs

In systemic sclerosis-associated ILD (SSc-ILD), telomere shortening has been directly linked to more severe pulmonary fibrosis and worse clinical outcomes, underscoring its role in disease progression [52]. While telomere biology in sarcoidosis is less well understood, evidence suggests that shortened telomeres may contribute to chronic granulomatous inflammation and fibrotic remodeling in advanced or progressive disease forms [53]. In anti-synthetase syndrome-associated ILD (ASSD-ILD) and myositis-associated ILD, although specific studies are lacking, it is plausible that telomere attrition exacerbates autoimmune-driven inflammation and fibrosis, amplifying disease severity. These findings emphasize the need for further research to clarify the role of telomere dysfunction and explore therapeutic strategies targeting telomere-related mechanisms, with the potential to address overlapping pathogenic processes and improve patient outcomes.

## 3. Surfactant-Related Genes

A surfactant is a complex mixture of lipids, phospholipids, fatty acids, and proteins that coats the inner surface of the alveoli. Its primary function is to reduce surface tension in the peripheral airways, thereby stabilizing the alveoli and preventing collapse during expiration. Surfactant is produced by type II pneumocytes, where its components are stored within lamellar bodies in the endoplasmic reticulum and secreted into the airways through exocytosis [54]. Once in the airways, a surfactant is either recycled by type II cells or catabolized by alveolar macrophages, maintaining the system’s homeostasis [55]. Beyond its role in maintaining alveolar stability, surfactant also serves as a key player in immune defense, regulating both immune responses and lung inflammation. Surfactant proteins constitute up to 10% of surfactant weight and include four types: SP-A1 and A2, SP-B, SP-C, and SP-D, each encoded by their respective genes—SFTPA1, SFTPA2, SFTPB, SFTPC, and SFTPD. The hydrophilic proteins SP-A and SP-D play defensive roles by interacting with various pathogens, promoting their uptake, and regulating the activity of innate and adaptive immune cells such as macrophages, dendritic cells, neutrophils, and T cells [56]. In contrast, the hydrophobic proteins SP-B and SP-C are involved in the mechanical functions of surfactants, with SP-B being particularly crucial for the processing of SP-C within lamellar bodies. Mutations in SFTPA1, SFTPA2, SFTPB, and SFTPC—excluding SFTPD—are recognized as monogenic causes of interstitial lung disease. SFTPB mutations are most associated with severe respiratory failure in newborns, while mutations in the other surfactant-related genes are implicated in both adult and pediatric forms of interstitial lung disease, particularly pulmonary fibrosis [54]. Additionally, mutations in genes involved in the transport of surfactant lipids within lamellar bodies, such as ABCA3 (ATP binding cassette subfamily A member 3), and in regulatory genes such as NKX2-1 (encoding the thyroid transcription factor 1, TTF1), which controls the transcription of surfactant proteins and ABCA3, have also been identified [8]; therefore, disruptions in the factors responsible for surfactant system homeostasis are now recognized as monogenic causes of interstitial lung disease [54].

### 3.1. Mutations in SFTPC and ABCA3 in FPF

In major cohorts of patients with FPF, mutations in surfactant-related genes rank second, contributing up to 8% of genetic involvement, following telomere-related alterations. To date, at least 26 mutations in SFTPC (up to 3.6%) have been identified in FPF patients, all presenting in heterozygosity and transmitted in an autosomal dominant manner [54]. These mutations often manifest as single nucleotide variants, predominantly occurring in the C-terminal BRICHOS domain of the pro-protein SP-C. This domain plays a crucial role in the processing and proper folding of pro-SPC [57]. Such mutations have been primarily observed in unrelated children presenting with severe acute respiratory failure at birth. Among these, the I73T mutation—where threonine replaces isoleucine at codon 73—is the most well-known. In a study conducted by Bullard et al., the genomes of 325 children were analyzed, revealing 55 carriers of SFTPC mutations, 19 of whom harbored the I73T variant. This substitution was associated with a broad spectrum of respiratory manifestations, ranging from neonatal death to the onset of symptoms within a few months of life or the development of pulmonary fibrosis in the fifth to sixth decade [58]. In a study led by Van Moorsel, the genetic contributions of SFTPC and ABCA3 were examined in a cohort of 229 patients with IIP. Among 20 unrelated adult FPF patients, SFTPC sequencing revealed mutations in five cases, with I73T being the most frequent. Two other cases involved previously unknown mutations, M71V and IVS412. Notably, no mutations were identified in the control cohort or among patients with sporadic pulmonary fibrosis (*n* = 20). Furthermore, some FPF patients were found to harbor ABCA3 variants [59]. The ABCA3 gene encodes a protein localized to the limiting membrane of lamellar bodies, where it is essential for surfactant storage and lamellar body biogenesis. Mutations in ABCA3, such as the autosomal recessive E292V variant, result in severe neonatal acute respiratory distress syndrome (ARDS) or adult-onset interstitial lung disease. The presence of ABCA3 mutations appears to modulate disease severity and increase penetrance in individuals with the same sporadic SFTPC mutation [58].

In 2011, a study of a Japanese family spanning three generations and comprising six individuals affected by pulmonary fibrosis with a UIP pattern led to the discovery of a significant mutation in the SP-C promoter, termed G100S. This mutation was found to induce endoplasmic reticulum stress within pneumocytes, leading to apoptosis in affected individuals [60]. Additionally, mutations affecting the SP-C promoter have been associated with its degradation, resulting in a deficit or absence of SP-C, which likely contributes to the pathogenesis of pulmonary fibrosis [58].

In contrast to familial forms of pulmonary fibrosis, the findings in sporadic cases have been less significant. A 2004 American study conducted genetic sequencing of SFTPC in 89 patients with a sporadic UIP pattern, 46 with an NSIP pattern, and 104 healthy controls. Although some genetic variants were identified in patients with IPF, only the I73T variant was found to result in a significant alteration of the surfactant protein [61]. Similarly, a 2007 German study analyzed SFTPC in 35 patients with sporadic IPF (25) and NSIP (10). This study identified only two allelic variants that led to the same amino acid change, yet these variants were found at comparable frequencies in healthy controls. As a result, de novo mutations in the SFTPC gene are considered rare contributors to the pathogenesis of sporadic pulmonary fibrosis [57].

### 3.2. Mutations in SFTPA1 and SFTPA2 in IPF and FPF

Although less frequently studied, mutations in surfactant protein A (SP-A) have also been investigated (Table 1). There are two isoforms, SP-A1 and SP-A2, with mutations in SFTPA1 being associated with endoplasmic reticulum stress. This stress can trigger a cellular response to misfolded proteins, leading to apoptosis and promoting epithelial-mesenchymal transformation, ultimately contributing to the development of pulmonary fibrosis [62]. Mutations in the gene encoding SP-A1 have been identified in patients from three Asian families with FPF, specifically affecting exon 6 [63]. Additionally, Takezaki and colleagues reported a homozygous mutation in two Japanese FPF patients, which resulted in decreased secretion of SP-A and increased susceptibility to influenza virus, potentially accelerating disease progression [64]. Wang and colleagues identified seven variants in exon 6 of the gene encoding SP-A2 in the sequencing of SFTPA2 from individuals in two families with pulmonary fibrosis [65]. A Dutch group replicated this sequencing in FPF patients, who were unrelated, as well as in patients with sporadic pulmonary fibrosis, excluding those with known mutations in SFTPC and telomere-related genes. They identified three novel variants of the N210T mutation in exon 6 among three FPF patients and one patient with sporadic pulmonary fibrosis. Exon 6 appears to be a critical region for SFTPA2 mutations, as it encodes a carbohydrate recognition domain involved in pathogen defense. Similar to SFTPC, mutations in SFTPA2 seem to induce endoplasmic reticulum stress, which is also expressed in club cells [66].

### 3.3. Mutations of SP Related Genes in RA-ILD

A multicenter French study conducted comprehensive genetic sequencing in patients with RA-ILD, focusing on genes linked to IPF, particularly SFTPC. This study identified mutations such as I73T and a mutation affecting intracellular transport in the SP-C promoter among RA patients. This study was the first to demonstrate that IPF and RA-associated ILD share common genetic mutations involved in their pathogenesis [9].

### 3.4. Mutations of SP Related Genes in CHP

Since HP involves an abnormal immune response to inhaled antigens, it has been hypothesized that alterations in surfactant proteins, which play a key role in immune defense and regulation, may contribute to its pathogenesis; however, research in this area remains limited, with only a few studies conducted on small populations, and the available evidence is not robust. The findings primarily focus on interactions between single SNV) and haplotypes in surfactant protein genes. A notable study was conducted in a Mexican population, including 75 HP patients with known antigen exposure, 64 exposed individuals without HP, and 194 unexposed healthy controls. Seventeen SNVs in surfactant proteins were analyzed, with a specific focus on the interaction between two or three SNVs and their potential impact on the risk of developing HP [67]. In the one- and two-SNV models, no significant association between surfactant protein gene SNVs and HP was observed when compared with exposed controls; however, in the three-SNV model, 25 significant interactions were identified, 16 of which were associated with an increased risk of HP. Furthermore, SNV interactions in hydrophilic surfactant protein genes such as SFTPA1, SFTPA2, and SFTPD were linked to an elevated risk of HP.

In 2022, the same research group expanded their analysis by studying SNV associations in the Mexican population, aiming to compare the genetic correlations with the development of similar fibrosing patterns in pulmonary fibrosis. This study involved 84 patients with IPF and 75 with HP. Among the SNVs analyzed, three in SFTPA1 and one in SFTPB were found to be associated with a decreased risk of IPF but an increased risk of HP. Many of the SNV-SNV interactions were shared between IPF and HP, although one three-SNV interaction involving SFTPA1, SFTPA2, and SFTPD was disease-specific, being linked to a decreased risk in HP and an increased risk in IPF [68].

### 3.5. Mutations of SP-Related Genes in Other ILDs

Although there is currently no direct evidence linking SP gene mutations to sarcoidosis, myositis-associated ILD, anti-synthetase syndrome-associated ILD, or SSc-ILD, their potential role in these conditions represents a promising avenue for future research. Indeed, mutations in SP genes disrupt surfactant production and processing, leading to alveolar epithelial cell dysfunction, endoplasmic reticulum stress, and impaired immune regulation—processes that overlap with the pathophysiology of these ILDs [54]. For instance, in sarcoidosis, SP dysfunction could theoretically contribute to persistent granulomatous inflammation and fibrosis. Similarly, in autoimmune-mediated ILDs, SP mutations might exacerbate chronic inflammation and fibrotic remodeling. In SSc-ILD, where alveolar epithelial injury is a hallmark, studying SP-related mechanisms could enhance understanding of fibrosis progression. While no direct associations have been identified, exploring SP gene mutations in these diseases may uncover novel insights into shared pathways of immune dysregulation and fibrosis, potentially leading to targeted therapeutic strategies.

## 4. MUC5B

Mucins are key components of mucus, a gel-like material composed of 97% water and 3% solids, primarily proteins, salts, and cellular debris [69]. The human genome contains 17 genes encoding mucins, with MUC5AC and MUC5B being the most prominently expressed in the airways [69]. MUC5B is located on chromosome 11 and encodes Mucin 5 subtype B, a glycoprotein secreted by submucosal glands and airway secretory cells [69]. This glycoprotein plays a crucial role in mucus production, mucociliary clearance, infection control, and maintaining homeostasis within the airways and lungs [70]. MUC5B is implicated in several pulmonary diseases, including chronic obstructive pulmonary disease (COPD), asthma, cystic fibrosis [71], and severe community-acquired pneumonia (CAP) [72]. A specific single nucleotide variant (SNV), rs35705950, located in the MUC5B promoter, is associated with increased promoter activity, leading to MUC5B overexpression [73]. The excessive production of MUC5B can result in its accumulation in the distal airways, impairing mucociliary clearance. This accumulation allows inhaled particles—such as those from cigarette smoke or air pollution, both known risk factors for IPF—to persist in the lungs, contributing to tissue injury. Furthermore, MUC5B overexpression and its accumulation in the distal airways may interfere with alveolar repair mechanisms by disrupting surfactant function and impairing type II epithelial cells [74]. This chain of events leads to lung injury, which in turn triggers chronic fibroproliferation and the formation of honeycomb cysts, characteristic features of progressive pulmonary fibrosis [75].

### 4.1. MUC5B in IPF and FPF

In 2011, Seibold et al. were the first to demonstrate that the T minor allele of the single nucleotide variant (SNV) rs35705950 in the promoter region of MUC5B is associated with an increased risk of developing both FPF and IPF [74]. The major allele of this SNV is guanine (G), while the minor allele is thymine (T) [74]. Subsequent studies confirmed the association between the MUC5B variant and sporadic IPF across multiple populations, including European Caucasian [76,77], American [78], and Chinese populations [79,80]; however, the frequency of the T minor allele varies among different populations. While it appears to be similar between American and European cohorts [74,76,77,78], its prevalence is significantly lower in the Chinese population [79] (Table 2).

Notably, the risk of developing IPF in carriers of rs35705950 is dose-dependent: homozygous individuals (with two copies of the T allele, genotype TT) face a higher risk of developing IPF compared with heterozygous individuals (with one copy of the T allele, genotype GT) [74,76,77,78,81] (Table 3). Moreover, Chung et al. found that the rs35705950 variant is linked to distinct HRCT patterns of fibrosis. Specifically, carriers of the T minor allele more frequently exhibit a UIP pattern with a subpleural distribution and are less likely to present ground glass opacities (GGO) compared with carriers of the G major allele [82].

The MUC5B variant is not only a genetic risk factor for IPF but also serves as a prognostic marker. Peljto et al. demonstrated in two independent cohorts of IPF patients that individuals carrying the GT and TT genotypes had a lower risk of mortality compared with those with the GG genotype [83]. The longer survival associated with the T allele was further confirmed in patients receiving antifibrotic therapy, regardless of whether the T allele was present in a homozygous or heterozygous form [75]. Additionally, Stock et al. reported a slower decline in forced vital capacity (FVC) in IPF patients with the MUC5B variant [76]; however, contrasting findings were reported by Jiang and colleagues, who observed in a Chinese cohort that the T allele was associated with worse survival outcomes, along with a greater decline in FVC and Diffusion of Lung Carbon Monoxide (DLCO) [80].

### 4.2. MUC5B in RA-ILD

In 2018, Juge et al. first demonstrated that the MUC5B promoter variant significantly increases the risk of developing ILD in patients with rheumatoid arthritis (RA), particularly in those with a UIP pattern [7]. This finding was later corroborated by Palomäki et al. in a Finnish study, which reported a tenfold increase in the risk of ILD among RA patients carrying the MUC5B variant compared with the general population [84]. Notably, Palomäki et al. also observed an increased risk of RA itself in individuals with the MUC5B variant, a relationship not identified by Juge et al., likely due to the smaller sample size in their study [7]. Further insights were provided by McDermott et al., who linked the MUC5B promoter variant to early onset RA-ILD, often manifesting before or within two years of RA diagnosis [85]; however, the MUC5B rs35705950 variant was not associated with the rate of pulmonary function decline in RA-ILD patients [86].

### 4.3. MUC5B in CHP

The MUC5B promoter variant is also associated with HP. A retrospective case-control study by Furusawa et al. demonstrated that common genetic risk factors for IPF, including the MUC5B variant, were also linked to the fibrotic form of HP [87]. Similarly, Ley et al. found that in patients with fibrotic HP, the MUC5B variant was associated with HRCT evidence of moderate-to-severe fibrosis and traction bronchiectasis [10]. Regarding the prognostic role of the MUC5B variant in HP patients, it has been shown that the variant is associated with poorer survival outcomes, a finding that contrasts with the survival advantage observed in IPF patients carrying the same variant [10]. Additionally, a recent study reported that carriers of the MUC5B minor allele had significantly lower baseline FVC, experienced a greater decline in FVC over time, and exhibited a worse response to immunosuppressive therapy compared with individuals with the GG genotype [88].

### 4.4. MUC5B in Other ILDs

The absence of an association between the MUC5B promoter variant and SSc-ILD has been observed in several studies conducted in both UK Caucasian populations [76,77] and an American cohort [83]. The lack of association between the MUC5B rs35705950 minor allele and lung fibrosis in SSc suggests that this variant may be specific to IPF or the UIP pattern. Notably, SSc-ILD is typically characterized by an NSIP pattern, while both IPF and RA-ILD are radiologically and pathologically defined by the UIP pattern. Supporting this, studies have demonstrated that in the lungs of IPF and RA-ILD patients, MUC5B expression is localized to areas of microscopic honeycombing [7,74]. The MUC5B variant is also not considered a risk factor in ASSD [89], myositis [90], or sarcoidosis [76]; however, the MUC5B rs35705950 promoter variant appears to be associated with asbestosis [11], potentially due to the fact that asbestosis shares radiological and pathological UIP features with IPF.

## 5. Toll-Interacting Protein

TOLLIP is a protein-coding gene that encodes a ubiquitin-binding protein involved in multiple aspects of the Toll-like receptor (TLR) signaling pathway. Four isoforms of TOLLIP (A, B, C, D) have been identified, with three expressed in human mononuclear cells, where they play a crucial role in innate immune responses and lung epithelial cell apoptosis. TOLLIP modulates the interleukin-1 beta (IL-1β) signaling pathway by inhibiting IRAK-1 (IL-1 receptor–associated kinase-1) autophosphorylation, promoting receptor degradation, and negatively regulating NF-kB activation [91,92]. Furthermore, it interferes with the release of IFN1 in respiratory tract cells. TOLLIP plays a central role in both acute and chronic inflammatory processes [93]. It is involved in immune-cell activation, cellular survival mechanisms, and defense against pathogens, particularly at the frontline of host defenses. Additionally, TOLLIP facilitates the clearance of damaged mitochondria through autophagosome-lysosome fusion [94,95]. By reducing mitochondrial reactive oxygen species and enhancing autophagy, TOLLIP suppresses mitochondrial apoptosis. As TOLLIP expression levels vary across cell types and disease stages, its downregulation accelerates apoptosis, contributing to early IPF development, while its upregulation in atypical epithelial cells drives fibrosis progression in late-stage IPF [95].

In pulmonary fibrosis, molecular mediators such as IL-6, MMP-7, and TGF-β secreted by lung epithelial cells exacerbate inflammation [96,97,98]. Given its role in inflammation regulation, autophagy, and vacuolar transport, TOLLIP is implicated in numerous pulmonary diseases, including IPF, COPD, asthma, and infectious diseases [95].

TOLLIP is ubiquitously expressed in lung cells (monocytes, macrophages, T-regulatory cells, and alveolar type 1 epithelial cells) in both healthy individuals and IPF patients [95]. Reduced or suppressed TOLLIP expression can lead to infectious and obstructive lung diseases by interfering with these cellular groups. Additionally, TOLLIP regulation may be altered by microRNAs, particularly the down-regulation of miR-31 (a tumor suppressor) in pulmonary diseases such as IPF [99].

In IPF, certain single nucleotide variants (SNVs) in the TOLLIP locus on chromosome 11 are linked to disease susceptibility and prognosis [100]. Specifically, the minor alleles G in SNV rs111521887, SNV rs5743894, and SNV rs5743890 correlate with reduced TOLLIP expression. The first two, in linkage disequilibrium, increase susceptibility to IPF but do not correlate with mortality, while rs5743890 appears to have a protective effect against IPF onset, although carriers exhibit a higher risk of rapid disease progression and mortality [95,100]. Moreover, the minor allele T of SNV rs3750920 is marginally associated with IPF susceptibility, progression, and survival, while SNV rs5743899 minor allele G is associated with other non-infectious pulmonary diseases (e.g., asthma) and infectious diseases such as tuberculosis and respiratory viral infections [95].

In CHP, the TOLLIP rs5743890 minor allele does not appear to be significantly associated with disease development compared with healthy individuals; however, patients with the TOLLIP rs5743899 GG genotype, associated with lower TOLLIP transcription-translation levels, exhibited rapid declines in FVC and increased Smad2 phosphorylation and inhibitor of NF-kB activity in lung tissues. Elevated serum levels of periostin, IL-1α, IL-1β, IL-6, IL-8, TNF-α, and IFN-γ were also observed, correlating with a significant annual decline in FVC in these patients [101]. A study by Kubbara et al. utilizing blood samples from the International Lung Disease database at the University of Minnesota evaluated the efficacy of pirfenidone in 56 IPF patients. The findings indicated that patients with TOLLIP rs5743890 genotypes CC and CT, along with TGF-β rs1800470, had improved survival with prolonged therapy [102]. In a post-hoc analysis of the PHANTER-IPF clinical trial, Oldham et al. reported that N-acetylcysteine (NAC) provided benefits to IPF patients carrying the TOLLIP rs3750920 TT genotype (present in approximately 25% of IPF patients), whereas patients with the CC genotype showed an increased risk of hospitalization. Nonetheless, genotype-stratified clinical trials are required before considering the off-label use of NAC in IPF treatment [103]. A study on Japanese patients with ILD found that the minor allele T of rs3750920 was less frequent in Japanese IPF patients compared with the European population from the PHANTER trial. Non-IPF patients exhibited an even lower prevalence of this allele, and those carrying the minor allele demonstrated better survival rates than non-carriers [104]. Finally, the PRECISIONS clinical trial (Prospective Treatment Efficacy in IPF Using Genotype for NAC Selection) has shown the potential for personalized medicine in IPF treatment, using molecular techniques to identify patients who may benefit from NAC therapy. This trial is currently in phase III, and further studies are required to confirm these findings [105].

The list of key studies on gene variants in ILDs, based on the updated literature, is presented in Table 4, while Table 5 provides the list of genes expressed by the different lung cell populations.

## 6. Epigenetic

In recent years, the critical role of epigenetics in regulating gene expression across various diseases has been increasingly recognized. In the context of pulmonary fibrosis, significant findings have emerged, particularly in IPF and secondary forms such as RA-ILD and SSc-ILD. Epigenetic modifications influence gene transcription by activating or silencing specific genes without altering the underlying DNA sequence. In pulmonary fibrosis, these modifications primarily affect genes encoding proteins involved in fibrogenesis signaling pathways [3].

Environmental factors, developmental processes, and aging are known to trigger these epigenetic changes. Among environmental influences, exposure to inhalants has been strongly linked to the development of IPF, with cigarette smoke being the most prominent risk factor for both IPF and FPF [106].

The main epigenetic modifications observed in pulmonary fibrosis include DNA methylation, histone modifications, and the regulation of gene expression by non-coding RNAs, such as microRNAs and long non-coding RNAs [107].

### 6.1. DNA Methylation

DNA methylation occurs at cytosine–guanine (C-G) sites, predominantly located in gene promoter regions, through the action of DNA methyltransferases (DNMTs), which add a methyl group to cytosine. This process leads to chromatin condensation, preventing the binding of the RNA polymerase complex to the promoter, thereby silencing gene expression. The degree of chromatin condensation induced by DNA methylation is directly linked to varying levels of gene expression. In general, hypermethylation of the promoter reduces target gene expression, while hypomethylation results in increased expression [3]. In IPF, genome-wide methylation studies have identified differentially methylated regions (DMRs) that are absent in healthy controls. These DMRs can be located within or outside the promoter regions of certain genes. Among the most studied genes are MUC5B, prostaglandin E receptor 2 (PTGER2), and THY1 cell surface antigen [3].

The MUC5B gene contains a highly conserved enhancer region, including the genetic variant rs35705950, which appears differentially methylated. This enhancer plays a crucial role in regulating MUC5B expression by dynamically binding the transcription factor FOXA2 and RNA polymerase II. Increased methylation of this enhancer region is associated with elevated MUC5B expression and the presence of the T allele at rs35705950, contributing to an increased risk of IPF [108]. Similarly, the PTGER2 promoter, which encodes the PGE2 receptor, has been found to be hypermethylated in IPF, leading to a reduction in PGE2 receptor expression. This results in resistance to prostaglandin E2, a molecule that normally regulates fibroblast homeostasis and apoptosis [109]. The THY1 promoter, which encodes a differentiation antigen of thymocytes, is also hypermethylated in IPF fibroblasts, leading to a loss of Thy-1 expression and abnormal myofibroblast differentiation [110,111]. Notably, Robinson and colleagues demonstrated that this process also occurs in healthy fibroblasts under hypoxic conditions [112].

Another molecular study identified additional significant DMRs in IPF involving genes such as CXCL10, CCL2, FOXP1, and miR21 [113]. In the context of FPF, the role of DNA methylation remains poorly understood; however, there is evidence suggesting that environmental exposure can influence the methylation of specific genes, potentially contributing to disease development in genetically predisposed individuals with FPF [3].

To date, there is no evidence supporting the involvement of this epigenetic mechanism in secondary forms of interstitial lung disease.

### 6.2. Histone Modifications

Histone modifications, such as DNA methylation, induce changes in chromatin conformation, resulting in varying levels of gene expression. Among these modifications, acetylation is particularly significant. It involves the addition of an acetyl group to the N-terminal acetyl-CoA of histones, leading to a relaxed chromatin state known as euchromatin, which permits gene transcription. In contrast, deacetylation, mediated by histone deacetylase (HDAC) enzymes, compacts chromatin into heterochromatin, thereby preventing gene expression.

In IPF, a marked imbalance in HDAC enzyme activity has been observed in the affected cells. Specifically, an abnormal increase in HDAC expression, particularly in classes I and II, has been detected in fibroblasts/myofibroblasts and bronchial basal cells. Conversely, alveolar type II epithelial cells show a depletion of HDAC enzymes attributable to endoplasmic reticulum stress, senescence, and apoptosis [114]. Key enzymatic alterations in IPF include upregulation of EP300, a histone acetyltransferase involved in cell proliferation and differentiation [3]; reduced activity of acetyltransferases (HATs), leading to decreased prostaglandin E2 (PGE2) expression due to diminished COX2 expression [107]; and reduced acetylation of histones H3 and H4 at the Thy-1 promoter, resulting in gene silencing, as well as at the Fas promoter, contributing to fibroblast resistance to apoptosis [107].

### 6.3. miRNAs

MicroRNAs (miRNAs) are non-coding RNAs composed of 15–27 nucleotides that bind to the 3′ end of messenger RNAs (mRNAs), leading to their inhibition or degradation and resulting in gene silencing. Each miRNA can regulate multiple genes, and each gene can be influenced by several miRNAs. In epigenetic studies on IPF, it has been shown that miRNAs can be differentially up- or downregulated during fibrogenesis, modulating key signaling pathways, such as the epithelial-mesenchymal transition (EMT) pathways (both Smad-dependent and non-Smad-dependent), the TGF-beta signaling pathway, the Wnt/beta-catenin pathway, and the PI3K-Akt pathway. Consequently, miRNAs can be categorized as either profibrotic or antifibrotic based on their activity [3]. In IPF, the most relevant overexpressed miRNAs include miR-21, miR-199-5p, miR-200c, and miR-34, while miR-29, miR-31, let-7a, and let-7d are downregulated [3,107]. Of these, miR-21 stands out as the primary profibrotic miRNA, with its expression directly induced by TGF-beta. It facilitates the EMT process and collagen deposition by inhibiting Smad7 in the Smad2/3 fibrotic pathway.

Among the antifibrotic miRNAs, the miR-29 family is the most significant, and its downregulation in IPF promotes fibrogenesis. These miRNAs play a role in apoptosis regulation and control of collagen and extracellular matrix (ECM) gene expression. Given that both miR-21 and miR-29 exert their effects primarily on fibroblasts by targeting Smad3, it has been hypothesized that the balance between these two miRNAs may decisively influence pulmonary fibrogenesis [115]. Elevated serum levels of specific miRNAs have been identified in IPF, with miR-21-5p being particularly relevant. This miRNA is associated with increased lung damage, reflected in altered FVC and radiological deterioration, particularly in rapidly progressing IPF [99,116,117,118]. In progressive forms of IPF, lung biopsy studies have identified overexpression of miR-302c, miR-423-5p, miR-210, miR-376c, and miR-185, which are linked to rapid disease progression, while lower levels of miR-423-3p are observed in more stable cases [107,118]; however, it is important to note that most studies have not focused on fibrotic foci, which are central to the pathogenesis of IPF. In a study by Sabater and colleagues, miRNA expression in fibrotic foci was compared with that in fibroblast cultures and IPF-affected lung tissue. The results revealed significant differences and some overlap in the composition of miRNAs [119].

Dissimilar to IPF, few studies have investigated miRNAs in familial forms of pulmonary fibrosis (FPF). It is hypothesized that miRNAs, along with environmental exposures, may influence the onset and severity of pulmonary fibrosis in genetically predisposed individuals with FPF [3]. In secondary forms of pulmonary fibrosis, miRNAs have been extensively studied in systemic sclerosis-associated interstitial lung disease (SSc-ILD). Comparisons between IPF and SSc-ILD have identified similarities, such as the overexpression of miR-21-5p and miR-92a-3p (profibrotic) and the downregulation of the miR-29 family, miR-26a-5p, and let-7d-5p (antifibrotic) [115,120]. In patients with SSc-ILD, elevated levels of miR-200c have been detected in peripheral blood mononuclear cells (PBMCs), particularly in those with more severe fibrotic patterns, suggesting a potential prognostic role for this miRNA [121]. Additionally, miRNA-320a appears to regulate type I collagen expression. It is downregulated in the serum and PBMCs of patients with SSc-ILD but is upregulated by TGF-beta [122]. In RA-ILD, studies have shown significantly higher plasma levels of hsa-miR-214-5p and hsa-miR-7-5p in patients with RA-ILD compared with those with RA without ILD. This association is exclusive to patients with a UIP radiological pattern, and dissimilar to IPF patients, no increase in miR-21 levels was observed in RA-ILD patients [123].

### 6.4. Long Non-Coding RNAs (LncRNAs)

Among non-coding RNAs, long non-coding RNAs (lncRNAs) play a crucial role in mediating RNA–RNA, RNA–protein, and RNA–DNA interactions, as well as in regulating gene expression at both the transcriptional and post-transcriptional levels. LncRNAs are classified based on their proximity to protein-coding genes and their regulatory functions. Specifically, lncRNAs are categorized as *cis* if they regulate the expression of nearby genes and *trans* if they leave the transcriptional site and perform their function elsewhere within the cell. LncRNAs can also induce direct modifications, such as phosphorylation, or act as competitive inhibitors of specific miRNAs, thereby regulating the expression of their target genes [124]. In pulmonary fibrosis, several studies, some ongoing, have revealed promising results, particularly in IPF and SSc-ILD. In IPF, the two most studied lncRNAs involved in the fibrogenesis process are DNM3OS and FENDRR [3]. 

DNM3OS is a fibroblast-specific lncRNA that regulates the activation of myofibroblasts induced by TGF-beta through certain profibrotic miRNAs, including miR-199a-5p/3p and miR-214-3p, within both Smad-dependent and non-Smad-dependent TGF-beta signaling pathways [125]. Conversely, FENDRR is a lncRNA that is downregulated in IPF fibroblasts and functions to inhibit fibroblast activation through multiple mechanisms, including reducing iron concentration by interacting with IRP1 and competing with miR-214, a known profibrotic miRNA [126]. In SSc, lncRNAs have been observed to modulate various pathogenetic phases, particularly the fibrotic phase. Among the lncRNAs studied, those most noteworthy for their roles in fibrogenesis include TSIX, H19X, HOTAIR, CTBP1, AGAP1, and ncRNA00201. TSIX influences chromatin remodeling and gene silencing, thereby affecting the expression of genes involved in extracellular matrix production. H19X contributes to fibrogenesis by acting as a molecular sponge for microRNAs that suppress pro-fibrotic pathways, leading to increased fibroblast activation and collagen synthesis. HOTAIR facilitates fibrosis by recruiting polycomb repressive complex 2 (PRC2) to target genes, inducing epigenetic modifications that promote myofibroblast differentiation. CTBP1 and AGAP1 regulate transcription factors and signaling pathways critical for fibroblast proliferation and extracellular matrix deposition, while ncRNA00201 interacts with pro-fibrotic signaling cascades, such as TGF-β, to amplify the fibrotic response [124].

## 7. Conclusions

Genetics and epigenetics have significantly advanced our understanding of the etiology and progression of both idiopathic and non-idiopathic ILDs. Key genetic factors, such as TRG mutations, SP variants, and the *MUC5B* promoter variant, have been linked to disease susceptibility, earlier onset, and more severe prognoses. These genetic pathways often intersect with epigenetic modifications, further modulating disease risk, and prognosis through environmental triggers. Despite these advancements, there remains no universally accepted genetic panel to guide the identification of relevant mutations in individuals with a family history of pulmonary fibrosis, and the impact of these genetic factors on treatment responses is largely unexplored. Addressing this gap represents a critical area for future research. Understanding how these genetic and epigenetic alterations influence therapeutic outcomes has the potential to drive the development of precision medicine approaches, ultimately improving individualized care and outcomes for ILD patients.

## Figures and Tables

**Table 1:** Mutations and variants associated with interstitial lung diseases.

			IPF		RA-ILD	cHP	HP
			s-IPF	f-IPF			
Mutations	Telomers	TERT	**+**	**++**	**+**	**+**	
		TERC	**+**	**++**			
		RTEL1	**+**	**++**	**+**	**+**	
		PARN	**+**	**++**	**+**	**+**	
	Surfactants	SP-A1		**+**		**+**	
		SP-A2	**-**	**+**		**+**	
		SP-C	**+**	**++** *I73T mutation*	**+** *I73T mutation*		
		SP-D				**+**	
		ABCA3		**+** *E292V mutation*			
Variants (SNVs)	MUC5B	rs35705950 *minor allele T*	**+** *genotype TT*	**+**	**+**		**+**
	TOLLIP	rs111521887 *minor allele G*	**+**				
		rs5743894 *minor allele G*	**+**				
		rs5743890 *minor allele G*	**+**				
		rs3750920 *minor allele T*	**+**				
		rs5743899 *minor allele G*				**+** *genotype GG*	

The table summarizes the most frequent gene mutations and variants associated with ILDs. +: positive association; ++: strong positive association; -: weak association. ABCA3: ATP binding cassette subfamily A member 3; CHP: chronic hypersensitivity pneumonitis; HP: Hypersensitivity pneumonitis; IPF: idiopathic pulmonary fibrosis; f-IPF: familial idiopathic pulmonary fibrosis; s-IPF: sporadic- idiopathic pulmonary fibrosis; MUC5B: Mucin 5B; PARN: Poly (A)-specific ribonuclease; RA-ILD: Rheumatoid arthritis interstitial lung disease; RTEL1: regulator of telomere length 1; SNV: single nucleotide variant; SP-A1: surfactant proteins A1 encoded by genes SFTPA1; SP-A2: surfactant proteins A2 encoded by genes SFTPA2; SP-C: surfactant proteins C encoded by genes SFTPC; SSc-ILD: Systemic Sclerosis interstitial lung disease; TERC: telomerase RNA component; TERT: telomerase reverse transcriptase; TOLLIP: Toll-interacting protein.

**Table 2:** Frequency of the T minor allele.

	IPF	CONTROL	FPF
Seibold et al. (American population) [74]	38%	9%	34%
Zhang et al. (American population) [78]	34.3%	11.1%	
Stock et al. (European UK population) [76]	36%	10%	
Borie et al. (European French and Italian population) [77]	41.9%	10.8%	
Wang et al. (Chinese population) [79]	3.33%	0.66%	

The table presents the frequency of the T minor allele across different populations and ethnicities.

**Table 3:** Odds Ratio for IPF based on MUC5B Variant of Allele T.

	Genotype TT	Genotype GT
Seibold et al. [74]	21.8 (95% CI, 5.1 to 93.5)	9.0 (95% CI, 6.2 to 13.1)
Zhang et al. [78]	9.7 (95% CI, 4.7 to 19.9)	5.9 (95% CI, 4.4 to 7.8)
Stock et al. [76]	11.81 (95% CI, 4.26 to 33.72)	6.62 (95% CI, 4.10 to 10.67)
Wu et al. [81]	10.12 (95% CI, 7.06 to 14.49)	4.84 (95% CI, 3.85 to 6.08
Borie et al. [77]	21.7 (95% CI, 10.4 to 45.3)	6.3 (95% CI, 4.6 to 8.7)

Probability of IPF based on homozygous and heterozygous states for the T genotype of MUC5B.

**Table 4:** List of key studies on gene variants in ILDs.

Study	ILD	Genes Analysed
Armanios et al. [31]	f-IPF s-IPF	TERT, TERC, RTEL1 and PARN TERT, TERC
Alder et al. [19]	f-IPF	TERT, TERC, RTEL1 and PARC
Tsakiri et al. [34]	s-IPF	TERT
Juge et al. [9]	RA-ILD	TERT, RTEL1, PARN, TERC and DKC1
Kannengiesser et al. [30], Vannier et al. [40]	f-IPF	RTEL1
Ley et al. [51]	CHP	TERT, TERC, RTEL1, and PARN
van Moorsel et al. [59], Bullard et al. [58]	FPF	ABCA3 variants
Doubková et al. [63], Takezaki et al. [64]	FPF	SP-A1
Wang, Y. et al. [65] and Van Moorsel et al. [66]	FPF and s-IPF	SP-A2
Juge et al. [9]	RA-ILD	SP-C
Gandhi et al. [67] and Abbasi et al. [68]	HP and IPF	SP-A1, SP-A2 and SP-D
Seibold et al. [74]	IPF and FPF	MUC5B rs35705950 minor allele T
Stock et al. [76]	IPF, SSc-ILD and sarcoidosis	MUC5B rs35705950 minor allele T
Borie et al. [77]	IPF and SSc-ILD	MUC5B rs35705950 minor allele T
Zhang et al. [78], Wang et al. [79], Jiang et al. [80]	s-IPF	MUC5B rs35705950 minor allele T, genotype TT
Juge et al. [7], Palomäki et al. [84], Juge et al. [86]	RA-ILD	MUC5B rs35705950 minor allele T
Furusawa et al. [87], Ley et al. [10]	IPF and CHP	MUC5B rs35705950 minor allele T
Peljto et al. [83]	SSc-ILD	MUC5B rs35705950 minor allele T
López-Mejías et al. [89]	ASSD	MUC5B rs35705950 minor allele T
Johnson et al. [90]	autoimmune myositis	MUC5B rs35705950 minor allele T
Platenburg et al. [11]	IPF and asbestosis	MUC5B rs35705950 minor allele T
Li, X. et al. [95], Noth, I. et al. [100]	IPF	TOLLIP alleles G in SNV rs111521887, SNV rs5743894, SNV rs5743890 and minor allele T of SNV rs3750920
Katayanagi, S. et al. [101]	CHP	TOLLIP rs5743890 minor allele and rs5743899 GG genotype

The table summarizes key studies on gene variants in ILDs, highlighting the ILD types and the relevant genes analyzed in each study. ABCA3: ATP binding cassette subfamily A member 3; ASSD: anti-synthetase syndrome; CHP: chronic hypersensitivity pneumonitis; HP: Hypersensitivity pneumonitis; IPF: idiopathic pulmonary fibrosis; f-IPF: familial idiopathic pulmonary fibrosis; s-IPF: sporadic idiopathic pulmonary fibrosis; FPF: familial pulmonary fibrosis; lncRNAs: long non-coding; miRNAs: microRNAs; MUC5B: Mucin 5B; PARN: Poly (A)-specific ribonuclease; RA-ILD: Rheumatoid arthritis interstitial lung disease; RTEL1: regulator of telomere length 1; SP-A1: surfactant proteins A1 encoded by genes SFTPA1; SP-A2: surfactant proteins A2 encoded by genes SFTPA2; SP-C: surfactant proteins C encoded by genes SFTPC; SSc: Systemic Sclerosis; SSc-ILD: Systemic Sclerosis interstitial lung disease; TERC: telomerase RNA component; TERT: telomerase reverse transcriptase; TOLLIP: Toll-interacting protein.

**Table 5:** List of genes expressed by different lung cell populations.

Genes Family	Genes Names	Expressing Lung Cells
Telomere-related genes	TERT, TERC PARN	Stem cells, progenitors, Fibroblasts
Surfactant-related genes	SFTPA1, SFTPA2, SFTPB, SFTPC, SFTPD.	alveolar type 2 epithelial cells
Toll-interacting protein	TOLLIP (A, B, C, D)	monocytes, macrophages, T-regulatory cells, and alveolar type 1 epithelial cells
Mucins	MUC5B, MUC5AC	Airway secretory cells

The table presents a list of genes expressed in different cell populations within the normal lungTERT: telomerase reverse transcriptase, TERC: telomerase RNA component; surfactant proteins, PARN: Poly (A)-specific ribonuclease; A1:SFTPA1; surfactant proteins A2:SFTPA2; surfactant proteins C: SFTPC; TOLLIP: Toll-interacting protein. MUC5B: Mucin 5B.
